# Three-Dimensional Architecture and Mechanical Properties of Bovine Bone Mixed with Autologous Platelet Liquid, Blood, or Physiological Water: An In Vitro Study

**DOI:** 10.3390/ijms19041230

**Published:** 2018-04-18

**Authors:** Antonio Scarano, Francesco Inchingolo, Giovanna Murmura, Tonino Traini, Adriano Piattelli, Felice Lorusso

**Affiliations:** 1 Department of Medical, Oral and Biotechnological Sciences and CeSi-MeT, University of Chieti-Pescara, via dei Vestini 31, 66100 Chieti, Italy; giovannamurmura@gmail.com (G.M.); t.traini@gmail.com (T.T.); drlorussofelice@gmail.com (F.L.); 2 Department of Interdisciplinary Medicine, University of Bari “Aldo Moro”, 70121 Bari, Italy; francesco.inchingolo@uniba.it; 3 Department of Medical, Oral and Biotechnological Sciences, University of Chieti-Pescara, via dei Vestini 31, 66100 Chieti, Italy; apiattelli@unich.it

**Keywords:** autologous platelet, bovine bone, biomaterial, sticky graft, biomaterials, fibrin

## Abstract

In recent years, several techniques and material options have been investigated and developed for bone defect repair and regeneration. The progress in studies of composite graft materials and autologous platelet-derived growth factors for bone regeneration in dentistry and their biological and biomechanical properties has improved clinical strategies and results. The aim of this study was to evaluate the three-dimensional architecture and mechanical properties of three different combinations of composite bovine graft, adding autologous platelet liquid (APL), blood, or physiological water. One experimental group for each combination of biomaterials was created. In particular, in Group I, the bovine graft was mixed with APL; in Group II, it was mixed with blood, and in Group III, the biomaterial graft was combined with physiological water. Then, the composite biomaterials were evaluated by scanning electron microscopy (SEM), and a compression-loading test was conducted. The evaluation showed a statistical significance (*p* < 0.01) of the elastic regime of deformation resistance, in which the combination of APL with bone graft resulted in an 875% increase in the mechanical resistance. The protocol of APL mixed with bovine bone graft produced a composite sticky graft block that was capable of increasing the mechanical properties in order to improve its clinical use in the treatment of the maxillary bone defects.

## 1. Introduction

The use of biomaterials has been increasing in clinical practice. They are available in different shapes and sizes and in unlimited amounts, but they require longer healing periods in comparison to autologous bone due to the reduced biological potential, as they are cell-free [1]. Currently, bone substitute materials are considered a valid alternative; however, compared with autologous bone, they show smaller areas of regenerated bone [2]. Numerous studies have been dedicated to improving the performance of different biomaterials for bone regeneration. Various synthetic or biological materials have been used for bone regeneration, including: autologous bone; demineralized and mineralized freeze-dried allografts; anorganic bovine bone; anorganic porcine bone; collagenated porcine bone, coralline calcium carbonate; bioglass; polylactide/polyglycolide materials; synthetic polymers; calcium sulfate; and hydroxyapatite [2]. These are commercially available in cement pastes, granules, gel, and blocks of osteoconductive and osteoinductive biomaterials that have been investigated for bone reconstruction and augmentation. The ideal bone grafting material should have both osteoinductive and osteoconductive properties, and should also have a good mechanical resistance in occlusal loads and be easy to apply in clinical situations. Autologous bone is considered the “gold standard”, promoting angiogenesis, osteoinduction, osteoconduction, and osteogenesis, without the risk of disease transmission [3]. However, the use of autologous bone has some disadvantages, such as increased morbidity associated with a second surgical procedure, and limited availability [4].

When using the different biomaterials, it is advisable to know their physical characteristics, as this should facilitate their clinical application. To use them properly, a clear understanding of graft physiology is required.

The grafting materials that are utilized in bone regeneration help to maintain space between the bone and the periostium or membrane. This function is performed very well by blocks, while the biomaterials in granules have a poor ability to maintain space [5]. Today, there are also bone graft alternatives and adjuvant in the form of bone substitutes and recombinant human growth factors [6]. These were used with success in the improvement of bone apposition to implants in the early healing stages before implant placement [7]. Also, autologous cancellous combined with autologous platelet enhanced bone regeneration in an in vivo critical-size cylindrical defect [8]. Also, the combination of collagen 1 and Platelet Rich Plasma (PRP) produced better bone healing in an in vivo pig model with a critical-size defect (10 mm × 8 mm, diameter × depth) in the forehead region [9].

Therefore, the use of scaffolds for cells and growth factor delivery has drawn a considerable amount of interest in bone restoration [10]. In the last decade, growth factors obtained from platelet concentrates have been used. Therefore, the use of biomaterials in particles turned into blocks using derivative platelets containing fibrin and growth factors is very interesting.

The aim of this study was to compare the architecture and mechanical properties of three different techniques for obtaining the moldable blocks of biomaterial through autologous platelet liquid, blood, or physiological water.

## 2. Results

### 2.1. Mechanical Characterization

**Figure 1:**
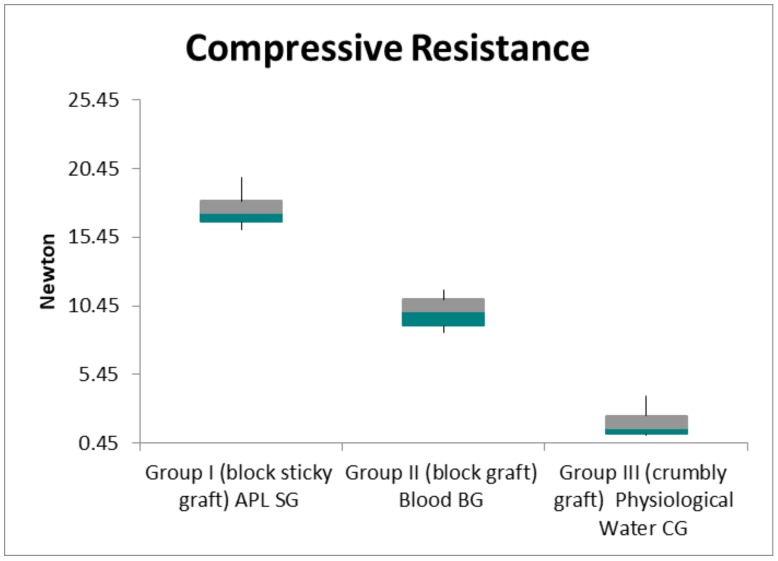
The values of a compressive test indicate a significant difference among the three groups. The Newton are expressed as mean ± SD.

**Figure 2:**
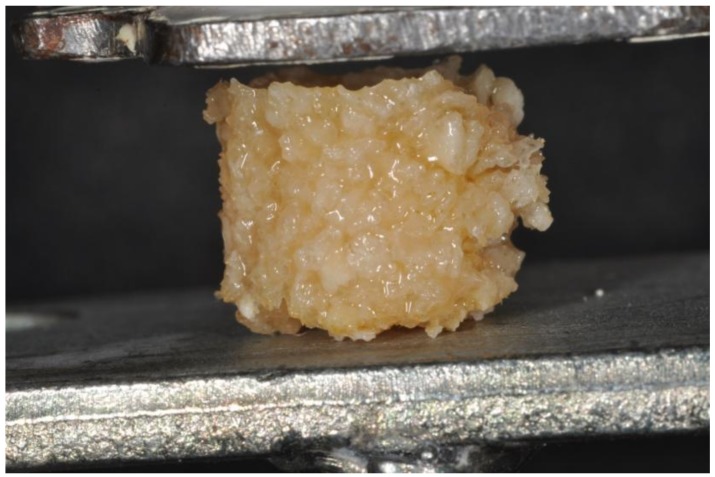
A cylinder of bovine bone mixed together with APL during the compressive test.

**Figure 3:**
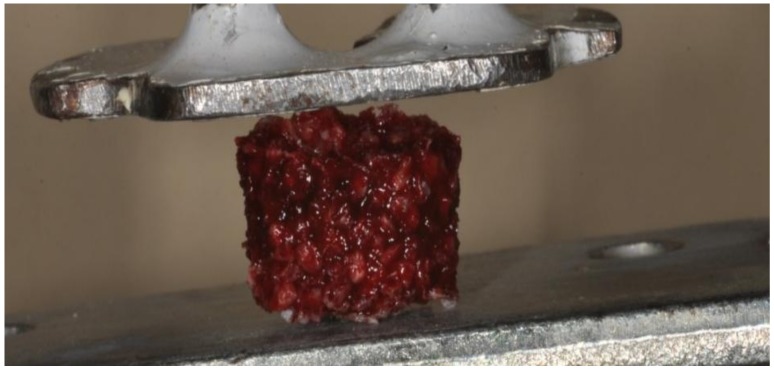
A cylinder of bovine bone mixed with blood during the compressive test.

**Figure 4:**
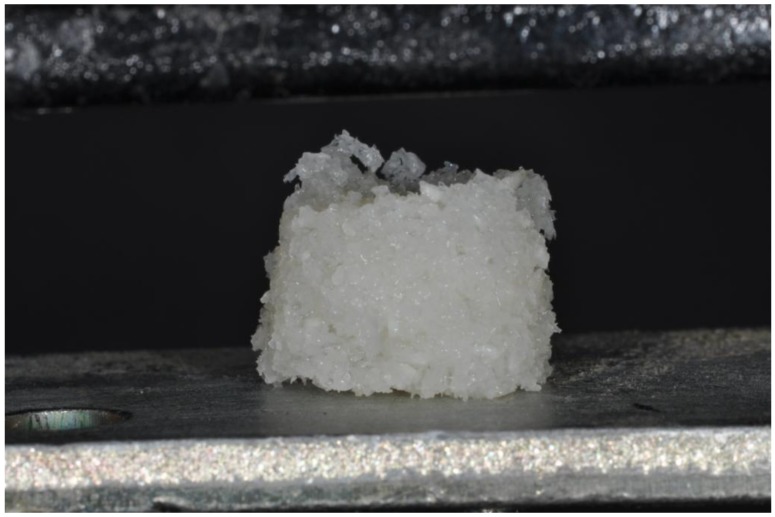
A cylinder of bovine bone mixed with physiological water during the compressive test.

**Figure 5:**
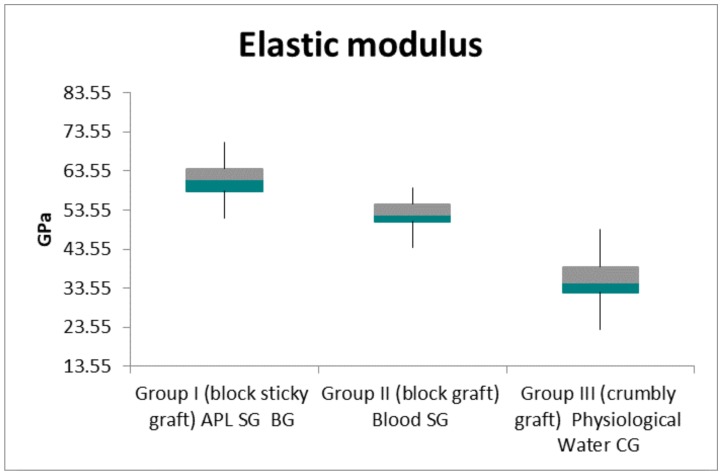
The graphic shows that the autologous platelet liquid (APL) (sticky bone graft) is capable of increasing the elastic modulus by 117.2% compared to the block graft (BG), and 178.7% compared to the crumbly graft (CG) group. The GPa are expressed as mean ± SD.

The data collected with the extensometer focused on the elastic regime of deformation of the 10 samples, and the calculated mechanical properties under compression (Table 1) showed that the sticky graft block was tougher than the other two samples. Group I (sticky graft block, or SG), Group II (block graft, or BG), and Group III (crumbly graft, or CG) showed a compressive resistance of 17.5 ± 1.3, 10.0 ± 1.1, *N* and 2.0 ± 1.1, respectively (Table 1; Figure 1). The specimens showed a linear range in which the stress increased in proportion to the strain (Figure 2, Figure 3 and Figure 4). The slope of this region was defined as Young’s modulus, or elastic modulus. The sticky graft block (E = 61.3 ± 5.3 GPa) and graft block (E = 52.3 ± 4.4 GPa) were the most elastic under compression, while the crumbly graft had little resistance (E = 34.3 ± 8.3 GPa) (Table 2; Figure 5).

A comparative analysis of the elastic regime of deformation resistance values between the BG group, the SG group, and the CG group showed a high statistical significance (*p* < 0.01), which was determined by using a one-way ANOVA test. The Bonferroni post hoc method at a 5% level of significance was also used to determine the location and magnitude of the significant differences between the experimental groups. The APL on the SG granules led to a composite scaffold with increased resistance. Table 1 shows the compressive stress values of the BG, SG, and CG groups, and statistical comparisons between the three groups.

### 2.2. Scanning Electron Microscopy

**Figure 6:**
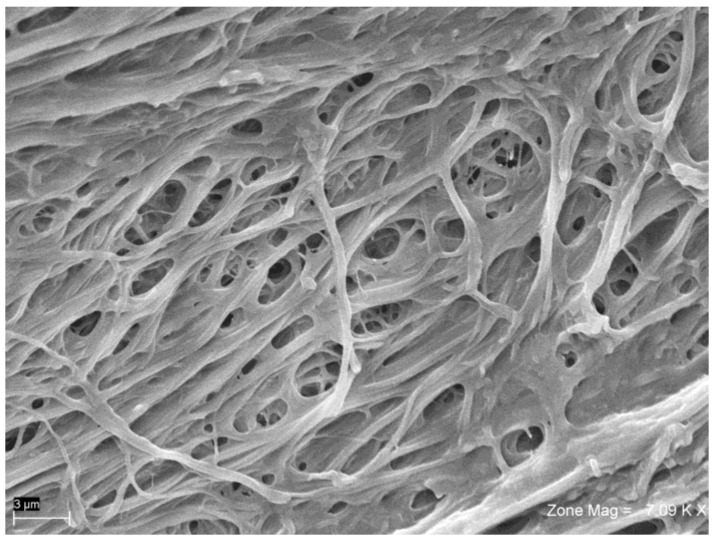
A granules of bovine bone mixed with APL covered by fibrin.

**Figure 7:**
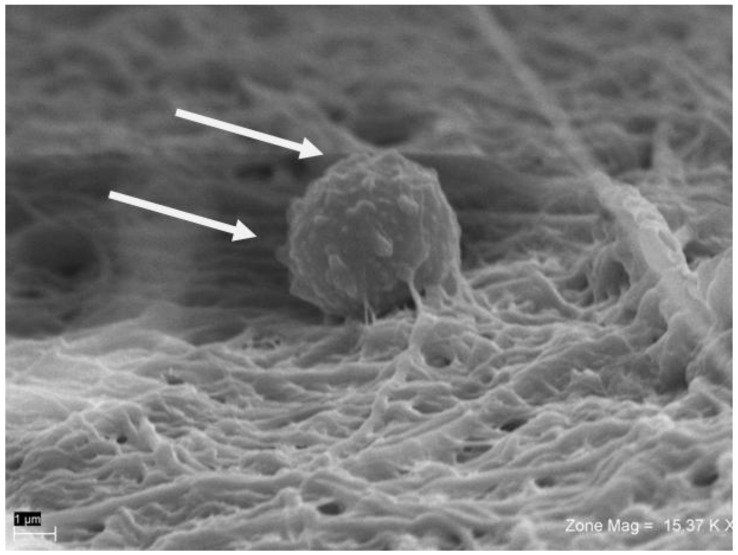
At higher magnification, it was possible to observe the structure of fibrin and activated platelets (Arrow).

**Figure 8:**
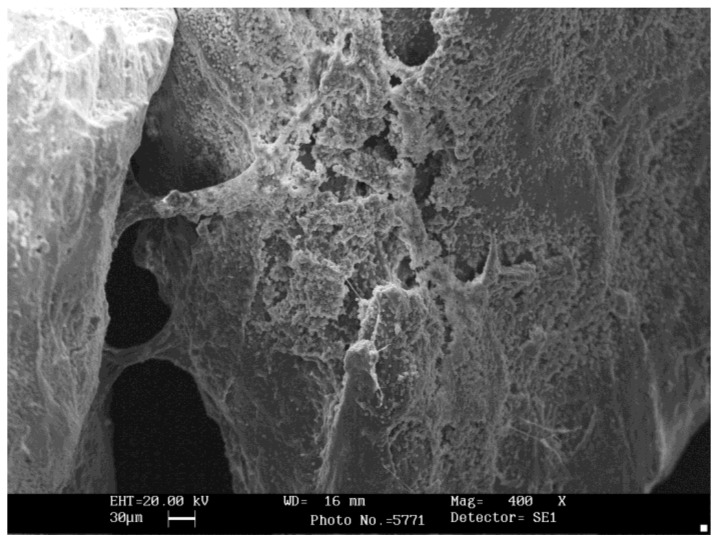
Scanning electron microscopy (SEM) of bovine bone mixed with blood. Many red blood cells and a thin layer of fibrin that only partially covered the biomaterial granules were observed; these appeared to be separated and not united.

Using SEM and back-scattered electron imaging, it was possible to observe a particle of bovine bone and blood cells (Figure 6, Figure 7 and Figure 8). The whole of each field was observed at low magnification, and any areas of interest showing contact with fibrin (Figure 6) and platelets were observed at high magnification (Figure 7). Sites of interest containing platelets were identified. All of the fields and particles analyzed contained predominantly fibrin (Figure 7), and very few platelets. The amount of fibrin fibers in bundle form was greater in the BG group than in the SG group.

The fibrin fiber network in the BG group was sparse, with many red blood cells and leucocytes, and a very loose fibrin matrix (Figure 7). The SG group showed the most condensed fibrin bundles (Figure 6), with platelet aggregates embedded within the fibrin network and in contact with biomaterials. In the SG group, bovine bone granules were cemented by fibrin, and platelets were found between bovine bone particles (Figure 7). Some SG samples also contained small amounts of cellular blood, but no red blood cells were observed. While in the BG group, many red blood cells were observed, as well as a thin layer of fibrin (Figure 8), which only partially covered the biomaterial granules and appeared separated and not united.

The number of adhered platelets on the bovine bone particles was 1.9 platelets per 1000 µm^2^ of area in the SG group, and 0.4 platelets in the BG group. In the CG group, the bovine bone granules showed structures of cancellous and cortical bone in a size range of 250–1000 µm, as shown in the label information. The granules were separated without an interposition of organic material.

## 3. Discussion

The results of the present study show that APL on SG granules lead to a composite scaffold with an increased compressive resistance of 175% compared with the value of the blood BG, and an increased compressive resistance of 875% compared with the saline solution crumbly graft group. Furthermore, the APL bone graft was capable of increasing the elastic modulus by 117.2% compared with the blood BG, and by 178.7% compared with the physiological water CG. This is important for the stability of bone grafts that are used for bone regeneration.

As a result of the experiments conducted in this study, we tested the three methods for producing a three-dimensional (3D) scaffold, to create a scaffold with autologous fibrin.

Different techniques were investigated to create a 3D scaffold, such as cross-linking agents with glutaraldehyde, a denatured form of collagen, poly (a-hydroxy acid) polyesters, poly-l-lactide (PLLA), poly-d-lactide (PDLA), EDC, and mTG [11]. In the present study, we used a platelet concentrate to produce a block for bone regeneration.

The applications of platelet concentrate have been increasing in different fields, such as dental implantology [12], orthopedic surgery [13], maxillofacial surgery [14], bone regeneration [15], oral surgery [16], dentistry, reconstructive surgery [17,18], and aesthetic medicine [19], since they were first proposed by Marx et al. [20], Anitua [21], and Choukroun [22] in the early 1990s.

The utility of fibrin glue [17] or platelet concentrates [23] during bone regeneration procedures is a current treatment concept that is used to accelerate hard and soft tissue healing and tissue maturation. Different protocols were proposed for producing a platelet concentrate such as APL, PRP, cPRP, PRF, PRG, PRGF, and platelet-rich fibrin [24]. These platelet concentrates were used for filling bone defects to accelerate the healing of bone or soft tissues [25].

In the present study, we evaluated the interaction of APL and bovine bone particles in order to create a sticky block of biomaterial. Interestingly, it has been demonstrated that a liquid platelet concentrate increases mechanical resistance and causes the formation of a sticky block when compared with bovine bone particles that were mixed only with blood or physiological water. The compressive test shows clearly that the SG group showed much higher values, while the CG group showed the lowest values. This can be because with the fibrin, the granules of biomaterials are pasted in areas that absorb extra strain, thus increasing the resistance to fracture. The rigidity of a biomaterial represents the material’s ability to resist deformation. Stiffness is commonly characterized by the slope of the linear region of a stress strain curve, which is also referred to as Young’s modulus, when tested under tension.

The outcome of the present study showed that mixing APL with bovine bone produces a flexible moldable sticky graft that has more mechanical strength. The mechanical property of the sticky graft itself is higher than bovine bone that is mixed with blood or physiological water. Thus, it is important to create a material that has a higher moldable rate and is mechanically stable under physiological conditions.

In this study, autologous platelets were used as the bonding agent to hold the granules of the biomaterials. The property of the sticky graft block allowed it to be easily placed in defects of any size or shape. In fact, the biomaterials that are used for bone regeneration must have appropriate mechanical properties for maintaining their shape against tensile forces and compression, and biocompatibility for interacting with host tissue and cells [26].

Biomaterials are manufactured in different forms such as blocks or granules in order to adapt to the various bone defect and clinical indications. The particles have poor mechanical properties, but are able to fill bone defects of different shapes and sizes, while the blocks have good mechanical properties, but must be modified in order to be placed into bone defects. In fact, the disadvantages encountered in the clinical use of granular biomaterials that are used to fill bone defects are: (1) migration near tissues; (2) low structural integrity of the scaffold; deformation and micromovement; and (3) collapse of the bone graft. A sticky graft reduces the migration of particles near tissues, and creates greater graft stability.

Other advantages of the sticky graft block technique include allowing the use of biomaterial particles to easily fill the bone defect. The use of particles of biomaterials instead of blocks could guarantee a better bone healing, since they have a higher osteoconductivity [27]. In fact, the geometry of their porous structure is an important aspect in the use of scaffolds, which encourages bone formation and facilitates mass transfer into pore networks [28]. The number of platelets and fibrin formation that we observed on the particles of the biomaterials that were mixed with APL may be due to the differences in the types of plasma proteins that were adsorbed. Another advantage of mixing APL with biomaterials is the creation of a network of fibrin, platelets, and growth factors.

The results indicated an increased mechanical resistance and increased percent of the surface that was covered by platelet adhesion. Another probable advantage is the growth factor that was present in the APL-imbibed biomaterial, which it slowly released. The growth factor works in concert and in a specific order to attract fibroblasts, which are inflammatory cells, as well as stimulate endothelial budding and collagen deposition. These outcomes lead to appropriate wound healing [28]. Angiogenesis and blood supply play an important role in bone formation [29]. Indeed, APL contains many growth factors in its naturally occurring and biologically determined ratio and is successful in acute wound healing. These growth factors include: platelet-derived growth factor (PDGF), vascular endothelial growth factor (VEGF), insulin-like growth factor-1 (IGF-1), epidermal growth factor (EGF), transforming growth factor-b1 (TGF-b1), basic fibroblast growth factor (bFGF), platelet activating factor (PAF), transforming growth factor a (TGF-a), coagulation (coagulation) factors, thrombospondin, platelet thromboplastin, serotonin, histamine, hydrolytic enzymes, and endostatin [30]. The mixing of graft material and platelet concentrate increases bone healing [31], and was used with success for treating peri-implant bone defects [32]. The combination of autologous platelet and biomaterial components has more potential for bone regeneration. In fact, the autologous platelet gels that were used combined with different biomaterials such as bioactive, bovine xenograft β-TCP, glass, or idrogel for better bone regeneration [9]. Platelet-derived growth factors influence a variety of cell–cell activities, serving as messengers and regulators in cell–extracellular and matrix interactions. Some of these bioactive molecules play a significant role in hard and soft tissue healing. Different studies, including in vivo animal studies, suggest that platelet-derivatives such as growth factors can be used to increase the healing of bone and soft tissue [28]. Our results demonstrated that the use of APL mixed with bovine bone in an in vitro study was more beneficial, as it facilitates handling and application. The outcome of the present study showed the possibility of producing a 3D scaffold consisting of granules of biomaterials with autologous platelets. Interconnected granule biomaterials are one of the key components in tissue engineering for bone healing, serving structural support to the healing tissue–cell interactions, as well as functioning as a carrier or template and as a bone extracellular matrix [33]. In this study, we chose to centrifuge the blood at 70 RCF for 15 min to produce the APL; this protocol avoids the second centrifugation. In fact, if we increase the G-force, we obtain gel. Other advantages were that lower centrifugation speeds were shown to contain higher numbers of cells, including leukocytes, prior to the formation of a fibrin clot; they also induced higher fibroblast migration and the expression of PDGF and TGF-β, and increased the concentrations of various growth factors and collagen 1 [34].

The inexpensive protocol used in this study enabled the relatively easily production of a block sticky graft. Additionally, APL with bovine bone facilitated the manipulation of its scaffolds and improved characteristics such as their mechanical properties, optimizing the scaffolds.

In conclusion, our results show that the APL mixed with bovine bone particles produced a 3D flexible, moldable sticky graft block; it also increased its mechanical resistance, and facilitated its clinical use when compared with bovine bone particles that were mixed only with blood or physiological water. These in vitro results help to set practice parameters for animal and clinical studies.

## 4. Materials and Methods

The study was ethically approved by the University of Chieti-Pescara Ethics Committee and has been registered as Clinical Trial number 2604, dated April 19th 2006. All patients gave written informed consent.

In the study, 10 patients (six males and four females, age ranging from 32 to 70 years, mean age 42 years) were enrolled between February 2007 and December 2017 to produce a total 40 samples.

The exclusion criteria were: severe systemic diseases (diabetes mellitus, gastrointestinal disorders, respiratory dysfunction, neoplasia, various carcinomas, etc.), smoking more than 10 cigarettes a day, pregnancy or lactating, history of antibiotics or non-steroidal anti-inflammatory drugs in the previous three months. The number of platelets of donors was 265,000 platelets/μL, (range from 20,000 to 350,000) taken from previous blood tests.

In clinical practice, blood is usually used for mixed biomaterials, or to produce autologous platelet liquid/gel and mixed biomaterials. For some cases of bone regenerative procedures, blood was used for our in vitro study to produce 20 samples of autologous platelet liquid for mixed biomaterials (Group I), and another 20 were mixed with biomaterials (Group II). In another group, the biomaterial was mixed with physiological water to produce 20 samples (Group III). A total of 60 samples were prepared: 30 for mechanical tests and 30 for SEM observations.

### 4.1. Autologous Platelet Liquid (APL) Preparation (Group I)

The blood of a healthy donor was collected as follows. Disposable kits were used. These kits for autologous platelet preparation and application, including: 4 mL × 9 mL blue vials with anticoagulant, 4 mL × 9 mL white vials for fractionation, 2 mL × 9 mL red vials with serum activator, 1 mL × 5 mL syringe, 1 mL × 1 mL activator syringe, 1 21-G butterfly needle for blood collection with a preassembled holder with a Luer lock attachment. Four white vials without anticoagulant or bovine thrombin were filled with the patient’s blood by venipuncture using a 21-G butterfly needle for blood collection with a preassembled holder with a Luer lock attachment. When the last tube was filled, the tourniquet and the needle from the patient’s arm was removed, using a swift backward motion. Gauze was immediately placed on the puncture site, and adequate pressure was applied to avoid the formation of a hematoma. After 1–2 min of pressure, a fresh piece of gauze or a Band-Aid was positioned on the puncture site. The vials were positioned in a counter-top device that was specifically designed for separating blood components. This is managed by a microprocessor that allows users to set the speed (RPM) or relative centrifugal force (RCF) and centrifugation time with the ability to customize programs. The centrifuge has a microprocessor that allows users to set the speed and have automatically the RCF value, and vice versa. The white vials were centrifuged at 70 RCF for 15 min at room temperature, producing some platelets and a fibrinogen-rich liquid.

According to the protocol, a 2.5-mL syringe was used to aspirate the layer that was rich in platelets [19]. Approximately 1 cc of autologous platelet liquid (APL) was produced per tube. The APL contained 6% RBCs, 93% platelets, and 1% white blood cells (WBCs), numerous amounts of fibrin strands, and water with blood protein. This suspension was mixed together with bovine bone (Re-Bone, UBGEN Padova, Italy) in a dappen glass and transferred to a cubic form of 1 cm × 1 cm. After 10 min, the mix solidified into a flexible, moldable sticky biomaterial (sticky graft block) (Figure 1). After preparation, 10 of the cubic imprint sticky grafts were observed under SEM, and another 10 were used for mechanical tests. The total preparation time from venipuncture to the production of flexible block sticky graft was approximately 27 min. The APL were mixed with bovine bone in a dappen glass, because the glass is a potent activator of platelets, and avoids the use of anticoagulants, bovine thrombin, or any other gelling agent [35].

### 4.2. Mix of Biomaterials and Blood Preparation (Group II)

One cc of blood was mixed with bovine bone with a size 0.25–1000 µm (Re-Bone, UBGEN Padova, Italy) in a dappen glass, and after 10 min was transferred to a cylinder form of 1 cm × 1 cm to produce a single cubic imprint. After 25 min, this mix solidified to produce blocks of biomaterial (block graft) (Figure 5). Ten of the cylinders of the imprinted blood and biomaterial mixes were observed under SEM, and another 10 were used for mechanical tests. The blood was mixed with bovine bone in a dappen glass, because the glass is a potent activator of platelets, and avoids the use of anticoagulants, bovine thrombin, or any other gelling agent [35].

### 4.3. Mix of Biomaterials and Physiological Water (Group III)

One cc of physiological water was mixed with bovine bone (Re-Bone, UBGEN Padova, Italy) in a dappen glass, and after 10 min was transferred to a cylinder form of 1 cm × 1 cm to produce a single cubic imprint. After 25 min, this mix solidified and produced a crumbly block biomaterial (crumbly graft) (Figure 2). Ten cylinder imprints were observed under SEM, and another 10 were used for mechanical tests.

### 4.4. Mechanical Investigations

A compression test was applied to determine the behavior of the cylinders under a compressive load. Compression tests were conducted by loading the test specimen between two plates, and then applying a force to the specimen. During the test, the specimen compressed, and the deformation versus the applied load was recorded. The compression test was used to determine the elastic limit and compressive strength. The mechanical properties of the investigated materials were characterized by using a static material testing device (Lloyd 30 K, Lloyd Instruments Ltd., Segensworth, UK) that was managed by a dedicated software (Nexigen, Batch Version 4.0 Issue 23, Lloyd Instruments Ltd.). Specifically, the compression was performed with a load applied to the samples with a constant crosshead speed of 1 mm/min to 10 cylinder imprints of blood and biomaterials (Group I), 10 cylinder imprints mixed with autologous platelet liquid and biomaterials (Group II), and 10 mixes of biomaterials and physiological water (Group III)

The load was applied parallel to cubic imprints (Figure 1, Figure 2 and Figure 5) The deformation load data were automatically recorded using Nexigen software (Nexigen, Batch Version 4.0, Issue 23, Lloyd Instruments Ltd.).

### 4.5. Scanning Electron Microscopy

Briefly, following blood incubation, samples were rinsed three times in phosphate-buffered saline (PBS) and fixed in 3% glutaraldehyde in PBS for 30 min, followed by 2% osmium tetroxide in PBS for 20 min, both at room temperature. Samples were subsequently dehydrated in a graded series of ethanol (from 30 to 96%). Out of absolute ethanol, the samples were left for 12 h in 113 Freon (trichlorotrifluoroethane) as a transition fluid to a critical drying point of CO2 (Tc 5 318C, Pc 5 73, 8 bar) using a critical-point dryer (Polaron CPD 7501 Bomb, Polaron Equipment, Watford, England). Finally, the samples were glued to aluminum stubs and coated with a very thin layer of gold (20 to 30 nm) by vacuum evaporation using a Techniques Hummer II-Au-sputtering (Techniques Inc., Chantilly, VA, USA).

The sample surface was examined with a scanning electron microscope operating at 20 to 30 KV, with tilt angles ranging from 10° to 45°. Scanning electron microscopy (SEM) back-scattered electron images were observed with a SEM (Cambridge Stereoscan 200, Cambridge Instrument Company Ltd., Cambridge, England.) SEM evaluations were performed by three independent observers who expressed an estimate of the amount of fibrin and platelets on the bovine bone particles. Five random areas that were 300 μm in diameter were evaluated for each sample, and an image in JPEG format was created. Quantization of the percentage of the biomaterial surface covered by fibrin and platelets was done on the JPEG images using a personal computer associated with a histometric software package with image-capturing capabilities.

### 4.6. Statistical Evaluation

A power analysis was performed using clinical software that was freely available on the site http://clincalc.com/stats/samplesize.aspx, in order to determine the number of specimens that were needed to achieve statistical significance for quantitative analyses of compression resistance and the quantization of the percentage of the biomaterial surface covered by fibrin. A calculation model was adopted for dichotomous variables (yes/no effect) by putting the effect incidence designed to caution the reasons as 10% for the controls and 95% for the treated substances. The optimal number of samples for analysis is 10 specimens for mechanical compression resistance, 10 for analyses of compression resistance, and 10 for the quantization of the percentage of the biomaterial surface covered by fibrin.

Differences between groups of treatment were analyzed by one-way analysis of variance (ANOVA) followed by Fisher’s protected least significant difference (PLSD) post hoc test. A *p*-value <0.05 was considered statistically significant. Statistical analysis was performed using the Statview software from SAS Institute (SAS Campus Drive, Cary North Carolina- USA).

## Figures and Tables

**Table 1:** Mean values and standard deviation (SD) of compressive resistance in the three groups.

Sample	Group I (Block Sticky Graft) APL SG	Group II (Block Graft) Blood BG	Group III (Crumbly Graft) Physiological Water CG
1	16.5	8.8	1.1
2	19.4	10.9	2.3
3	16.7	8.5	3.8
4	16.2	11.6	1.2
5	17.2	8.9	1
6	16	11	1.2
7	19.8	10.9	2.5
8	18.2	9.25	1.9
9	17.3	10.25	1.1
10	17.7	9.9	3.9
Compressive Resistance Mean	17.5 ± 1.3	10.0 ± 1.1	2.0 ± 1.1

**Table 2:** Mean values and standard deviation (SD) of elastic modulus in the three groups.

Sample	Group I (Block Sticky Graft) APL SG	Group II (Block Graft) Blood BG	Group III (Crumbly Graft) Physiological Water CG
1	70.9	56.1	36.8
2	51.4	59.3	39.7
3	65.5	49.2	32.5
4	57.3	43.1	22.2
5	63.6	50.2	34.1
6	59.1	55.7	39.9
7	61.5	52.1	32.7
8	64.3	52.9	48.8
9	58.1	52.6	20.5
10	61.3	51.8	35.8
Young Module Mean	61.3 ± 5.3	52.3 ± 4.4	34.3 ± 8.3
